# Toxicity and Tolerability of ^177^Lu-DOTA-TATE PRRT with a Modified Administered Activity Protocol in NETs of Variable Origin – A Phase 2 Registry Study

**DOI:** 10.2174/1874471014666210810100435

**Published:** 2022-04-27

**Authors:** Alireza Khatami, Golmehr Sistani, Duncan E. K. Sutherland, Sarah DeBrabandere, Robert H. Reid, David T. Laidley

**Affiliations:** 1 Division of Nuclear Medicine, Department of Medical Imaging, Western University, London, ON N645C1, Canada;; 2 Division of Radiology, Department of Medical Imaging, Western University, London, ON, Canada

**Keywords:** Toxicity, safety, neuroendocrine tumor, NET, PRRT, peptide receptor radionuclide therapy, DOTATAE, Lu-177

## Abstract

***Background*:** Peptide receptor radionuclide therapy (PRRT) has been recently approved for advanced, metastatic, or progressive neuroendocrine tumors (NETs).

***Objective*:** This study reports the adverse events (AEs) observed with patient-tailored administered activity.

***Methods*:** Fifty-two PRRT naive patients were treated with 177Lu-DOTATATE. The administered activity ranges between 2.78 and 5.55 GBq/cycle using the patient's unique characteristics (age, symptoms, blood work, and biomarkers).

***Results*:** The protocol was well tolerated with the overwhelming majority of participants being forty-six (88%), completing all 4 induction therapy cycles. The median cumulative administered activity was 19.6 GBq (ranged 3.8-22.3 GBq). A total of 42/52 (81%) reported at least one symptom, and 43/52 (83%) had evidence of biochemical abnormality at enrollment that would meet grade 1 or 2 criteria for AEs. These symptoms only slightly increase with treatment to 50/52 (96%) and 51/52 (98%), respectively. The most common symptoms were mild fatigue (62%), shortness of breath (50%), nausea (44%), abdominal pain (38%), and musculoskeletal pain (37%). The most common biomarker abnormalities were mild anemia (81%), reduced estimated glomerular filtration rate (eGFR) (58%), increased alkaline phosphatase (ALP) (50%), and leukopenia (37%). Of critical importance, no 177Lu-DOTATATE related grade 3 or 4 AEs were observed.

***Conclusion*:** Tailoring the administered activity of 177Lu-DOTATATE to the individual patient with a variety of NETs is both safe and well-tolerated. No patient developed severe grade 3 or 4 AEs. Most patients exhibit symptoms or biochemical abnormality before treatment and this only slightly worsens following induction therapy.

## 1 INTRODUCTION

Neuroendocrine tumors (NETs) are a diverse group of neoplasms that can involve all organs in the body. Even with similar NET histopathology, the clinical picture can be drastically altered with disease location, extent, and functional symptoms [1]. They are resistant to chemotherapy and have a high recurrence rate [2, 3]. However, with an indolent disease course, most patients can expect to maintain a good quality of life, even in a metastatic setting. The slower disease course of many raises patient expectations and drives the demand for low-toxicity treatment options.

The incidence of NETs in the USA is rising with a 4.8- fold increase over the last three decades from 1.09 to 5.25 /100,000 [4]. This rise is at least partly explained by advancements in diagnostic tools and improved physician awareness [5]. The 5-year survival of patients with NETs is 78% in localized disease and 27% in distant metastatic disease, highlighting the importance of early detection [6, 7]. NETs overexpress somatostatin receptors (SSTR). When somatostatin is bound to SSTR, these receptors gain antisecretory and antiproliferative effects that explain the mechanism of action of somatostatin analogs (SSAs), such as octreotide and lanreotide, which are used to slow disease progression and manage symptoms [8, 9].

Peptide receptor radionuclide therapy (PRRT) is a newer therapeutic option that demonstrates remarkable results in NET management and has an acceptable safety profile. PRRT therapies involve conjugating α or β particle-emitting radioisotopes to a somatostatin analog (SSA). This therapy enables a targeted treatment of somatostatin expressing NETs and spares otherwise healthy tissues. Thus, the toxicity profile is limited to tissue adjacent to tumors and organs with naturally high SSTR expression [10, 11].

The NETTER-1 trial reported four cycles of 7.4 GBq (200 mCi ) of ^177^Lu-DOTATATE separated by eight weeks per cycle prolonged progression-free survival in midgut NETs with a response rate approaching 1 in 5 patients [12]. They reported a low rate of serious AEs (grade 3-4), including neutropenia (1%), thrombocytopenia (2%), and lymphopenia (9%) [12]. A significant limitation of this study was enrollment limited only to midgut NETs. However, additional research supported the generalizability of PRRT therapy to other NETs [12-18]. The report presented herein uses a dynamic ^177^Lu-PRRT protocol that varies the administered activity according to the patient’s unique characteristics and clinical situation.

## 2 MATERIALS AND METHODS

### 2.1 Study Design

An ongoing, prospective single-center, single-arm open-label phase 2 registry study of ^177^Lu-DOTATATE treatment with a modified dose protocol started to recruit patients in 2014. ^177^Lu-DOTATATE used in this trial has been approved by Health Canada, and the study has been approved by our institutional Research Ethics Board (Ethics no: 104378).

The inclusion criteria were somatostatin receptor (SSRT) positive tumor on any SSRT scan (Krenning score 3 and 4), a Ki-67 < 20%, a life-expectancy > 26 weeks from enrollment, serum creatinine (Cr ) ≤ 130 μmol/L, creatinine clearance glomerular filtration rate (CrCl GFR) of ≥ 50mL/min/1.73 m^2^, hemoglobin (Hgb) concentration ≥ 90 g/L, white blood cells (WBC) ≥ 3 x 10^9^/L, absolute neutrophil count >1.5x10^9^/L, platelets (Plt) ≥ 100 x 10^9^/L, liver enzyme tests Alanine aminotransferase (ALT), Aspartate aminotransferase (AST) and alkaline phosphatase (ALP) ≤ 3 times the upper limit of normal, Eastern Cooperative Oncology Group scale Performance Status (ECOG PS) Score ≤ 2, 8) written informed consent before enrollment. Exclusion criteria included a history of previous PRRT, curative surgery, radiation therapy, radiofrequency ablation (RFA), other radioisotope therapy, cytotoxic chemotherapy, embolization, or other investigative therapy (interferons, mTOR inhibitors) within 12 weeks of enrollment, pregnancy, breastfeeding, uncontrolled diabetes mellitus, and other significant medical, psychiatric or surgical condition uncontrolled by treatment. Table (**1**) summarizes the inclusion/ exclusion criteria.

### 2.2 Treatment Protocol

As per study protocol, Sandostatin LAR was stopped 7 days before and for 7 days after ^177^Lu-DOTATATE therapy. Sandostatin (short-acting octreotide) was not administered 1 day before and 1 day after ^177^Lu-DOTATATE administration.

Participants were intended to be treated with four induction cycles of ^177^Lu-DOTATATE therapy every 8-12 weeks with administered activity ranged from 2.78-5.55 GBq (75-150 mCi). An IV line was inserted into an appropriate vein. Treatment started with an infusion of one liter of diluted amino acid solution (2.5% lysine and 2.5% arginine) one hour before ^177^Lu-DOTATATE administration. Then, ^177^Lu- DOTATATE was concomitantly injected with amino acid *via* a separate pump over 30 to 45 minutes. Finally, amino acid solution infusion continued (approximately 4 hours after the start of its infusion) until completed. Oral anti-emetic, granisetron, and dexamethasone were also administered up to 30 minutes before the amino acid infusion. After treatment, subjects were encouraged to drink plenty of fluids.

Amino acid solution and ^177^Lu-DOTATATE were provided by IDB Holland. ^177^Lu-DOTATATE was compounded locally. The amino acid solution and isotope were supported by Cancer Care Ontario.

After reviewing all patients’ information, potential subjects for ^177^Lu-DOTATATE therapy were reviewed at a multidisciplinary Neuroendocrine Tumor Board. Fifty-two patients signed a consent form once all eligibility criteria were met.

### 2.3 Assessment

All participants underwent a post-therapy scan following each treatment to evaluate the disease extent (typically at 24 hours). Those scans comprised total body planar imaging and single-photon emission computed tomography/ computed tomography (SPECT/CT). Those scans are used to assess uptake of the radiotracer in the primary and metastatic sites, response to treatment, or disease progression. Also, they required to have blood analysis after each treatment cycle, including estimated glomerular filtration rate (eGFR), Cr, ALP, AST, ALT, Hgb, Plt, WBC, and neutrophil counts before each ^177^Lu-DOTATATE therapy, on the day of treatment and weekly following each treatment up to 8 weeks and was visited in the clinic before next treatment. A pregnancy test for women participants of childbearing age before each treatment was also acquired. Any ongoing baseline symptoms or new AEs based on patients' self-reported symptoms at Edmonton Symptom Assessment System (ESAS) and ECOG PS in clinic visits and changes in biological markers and blood indices before and during our study were evaluated. All AEs were categorized according to the most current version of the National Cancer Institute’s Common Terminology Criteria for Adverse Events at the time of study design CTCAE 4.03 [19]. Based on this guideline, AEs grading 3 and 4 are considered severe and life-threatening that need hospitalization and urgent management, respectively. Grade 5 is a death related to treatment. Grade 1 and 2 AEs are mild and moderate and need observation or non-invasive intervention. Table (**2**) summarized the biomarker cut-offs for considering grade 3 and 4 AEs in this trial.

In our study, CrCl GFR was used for weekly eGFR measurements. However, for better assessment of renal function, we employed three methods for calculating eGFR, including CrCl (Cockcroft Gault equation), Modification of Diet in Renal Disease (MDRD) equation, and Chronic Kidney Disease Epidemiology Collaboration CKD-EPI equation at the screening, on treatment days, and designated visits thereafter before the next ^177^Lu-DOTATATE therapy.

Isotope GFR (iGFR) using Tc-99m-DTPA was used at baseline, 4-8 weeks after the second treatment, and the end of the fourth cycle as the gold standard for GFR measurement. Different methods of eGFR and iGFR and their correlation were assessed.

The interval of treatment (8-12 weeks) was advised upon clinical assessment for any AEs, results of the post-therapy scan, and blood works. Criteria of administered activity modification are provided in Table (**3**).

Upon the completion of 4 cycles of treatment, participants were reassessed. If the tumor responded to treatment or remained stable, then the patient entered the maintenance phase. This evaluation was scheduled approximately 6 months after the fourth induction therapy.

### 2.4 Statistical Analyses

All subjects who have received at least one treatment cycle of ^177^Lu-DOTATATE were evaluated for safety. Patients in the study also served as their own control. Descriptive analysis with a bar graph and swimmer plot were provided. Evaluation of the relationship between symptoms, hematologic indices, liver enzymes, and renal function values before and during/after treatment was performed using the McNemar test for paired nominal data and T-test for comparison of mean cumulative activity among different categories. Those analyses were done with SPSS version 25.

## 3 RESULTS

Fifty-two participants enrolled in this study between 2014 and 2017. Forty-six participants (88%) completed induction therapy (4 cycles), three (6%) completed two cycles, and an additional three (6%) completed only one cycle of PRRT therapy. Out of six participants who did not complete four cycles, three were withdrawn from the study due to cognitive decline and undisclosed memory impairment with radiation safety concerns, such as one who was incontinent and posed a radiation safety risk. Two participants withdrew consent after receiving the first administrated dose. Only one participant progressed on therapy after two cycles of treatment. The flow chart summarized our cohort. A total of 32/52 patients were male (62%). The patients were between 34 and 83 years old with a mean enrollment age of 63 years. 26/52 (50%) had midgut NET and 14/52 (27%) had pancreatic NET (PNET). Table (**4**) summarizes the cohort demographics, baseline characteristics, and anatomic sites of primary NETs. The median cumulative administered activity was 19.6 GBq (529.2 mCi) and ranged from 3.8 (102.7 mCi) in patients treated with only one cycle to 22.2 GBq (600 mCi) in patients who completed four cycles with maximal administered activity. 36/52 (69%) of participants received modification with reduced administered activity during induction therapy, including 31/46 (70%) who completed four-cycle therapy. The median cumulative activity among the cohort who completed four-cycle induction therapy was 20.6 GBq (556.7 mCi) and ranged from 14 to 22.5 GBq ( 378.4 – 608.1 mCi). 51/52 (98%) of participants categorized as a metastatic disease when enrolled in the trial and 1/52 (2%) had locally advanced disease with no distant metastatic disease. In 19/52 (36.5%) PRRT was the first treatment after initial diagnosis by surgery or disease progression on SSAs. However, 33/52 (63.5%) had a combination of chemotherapy, chemoembolization, radiotherapy, or RFA treatment before PRRT. 42/51 (82%) of metastatic NET patients demonstrated liver involvement. The median cumulative administered activity not significantly different between metastatic patients with liver and without liver involvement (19.1 GBq *vs*. 19.7 GBq) (516.2 *vs*. 532.4 mCi). Among the metastatic cohort, 45/51 (88%) completed four-cycle induction therapy with the median cumulative activity of 19.8 GBq (535.1 mCi), which was only slightly more than that of our cohort at 19.6 GBq (529.7 mCi). Fig. (**1**) demonstrates a Swimmer plot representing the cumulative administered activities of our cohort. The mean cumulative activity of the PNET group was higher than the midgut group but did not reach statistical significance (17.1 *vs*. 19.8, p= 0.1). Fig. (**2** ) demonstrates the cumulative activities among different primary NETs.

Forty-two (81%) of the participants reported at least one baseline symptom graded as 1 or 2 as per CTCAE. This number increased to 50/52 (96%) during the trial, representing an additional 8 patients (15%), and included ongoing baseline symptoms and new AEs. This finding met a prerequisite of statistical significance (p= 0.02). Importantly, none of the baseline or new symptoms were unexpected, serious, or life-threatening; all were categorized as grade 1 or 2 and responded to conservative management.

The most common reported symptoms during the trial were fatigue (62%), shortness of breath (50%), nausea (44%), abdominal pain (38%), musculoskeletal pain (37%), loss of appetite (35%), diarrhea (31%), flushing (29%) and headache (25%). Nausea, shortness of breath, and headache showed a correlation with ^177^Lu-DOTATATE therapy (p = 0.02, 0.013, and 0.004, respectively). The correlation between hair loss and ^177^Lu-DOTATATE therapy trended towards statistical significance (p = 0.06). The correlation between other symptoms and ^177^Lu-DOTATATE therapy was not significant.

Only 1 participant, 1/52 (2%), remained completely symptom-free throughout the study. Before the study, 32/52 (62%) participants reported ECOG PS ≥1. This number increased to 35/52 (67%) at the end of 4 cycles of therapy; however, this change did not meet statistical significance (p = 0.64). Reported symptoms and their incidences are summarized in Table (**5**).

Regarding blood variables, 43/52 (83%) had baseline grade 1 or 2 abnormalities before starting the study. By the end of the study, there had been an increase in blood variables AEs by 15% totaling 51/52 patients (98%) that met statistical significance (p= 0.008). The most commonly observed blood variables’ abnormalities before treatment were grade 1 or 2 anemia in 54% of patients, grade 1 eGFR reduction in 46% of patients, elevated Cr level in 8% of patients, and minimal hepatic injury with increased ALP, AST, and ALT in 25%, 6%, and 4% patients, respectively. As the trial progressed, we observed an increase in the frequency of these AEs. For example, the incidence of grade 1 anemia increased to 81%; grade 1 leucopenia and thrombocytopenia were observed in 37% and 25% of patients, respectively.

A mild rise of Cr in 15% with a concordant reduction of eGFR was seen in 58% of participants at the end of the fourth cycle of ^177^Lu-PRRT. The increase of grade 1 or 2 AEs was statistically significant for all blood variables except for neutrophil counts that had not met the criteria of significance. There were no severe or life-threatening grade 3 or 4 AEs reported in our study. No death related to ^177^Lu-DOTATATE therapy was observed.

Table (**6**) represents hematologic and biochemical abnormalities observed before trial and at the end of induction therapy.

Our data showed a mild variation in means of hematologic indices that steadily declined during the study. Hgb slowly declined to its lowest value between 4- and 8-weeks post-therapy. The same pattern is observed in other blood variables. The percentages of reduced indices at the end of the trial were, 5%, 29%, 29%, and 9% for Hgb, WBC, neutrophil, and Plt, respectively. There was a slight increase in serum Cr during the trial that did not reach statistical significance (p = 0.21).

Weekly eGFR shows an increase in grade 1 and 2 AEs from 46% of all participants to 58% by the end of the study. This trend also did not reach statistical significance (p > 0.05). iGFR demonstrated slightly better correlation with eGFR CKD-EPI than MDRD (baseline R2= 0.53 *vs.* R2= 0.5 and at the end of treatment R2= 0.64 *vs.* R2= 0.59). The correlation between iGFR and CrCl GFR was weaker (R2= 0.57 *vs*. R2=0.47 at baseline and the end of treatment, respectively). However, these variations did not alter categorization. The pattern of Cr changes demonstrated the highest values around eight weeks following each cycle. By the end of therapy, the mean Cr values increased by 9% above baseline. CrCl GFR followed a similar pattern with minimal fluctuation and a mild downward trend of 8.8% from baseline by the end of the study. The typical renal function pattern showed the lowest eGFR following the first cycle of treatment between weeks 2 and 7. Subsequent cycles demonstrate stable renal function following ^177^Lu-DOTATATE administration, likely from a combination of protocol tailored administered activity and reinforcing to the patient the importance of good hydration. No specific pattern for liver enzyme changes was observed. The ALT and AST reached their highest values toward the last weeks post each treatment cycle.

## 4 DISCUSSION

Patients with NETs may experience many symptoms as part of the natural history of their disease and their treatments. The most common clinical AEs in our cohort were fatigue (62%), shortness of breath (50%), nausea (44%), abdominal pain (38%), and musculoskeletal pain (37%). These numbers are consistent with those reported in the NETTER-1 trial [12] Table (**7**). The modified protocol of our study aims to tailor the patient characteristics to the administered activity to reduce toxicity. Our study found no severe or life-threatening AEs or patients withdrew from the trial due to treatment-related AEs Table (**8**). This is in contrast to the NETTER-1 trial with higher and fixed-dose, where 6% of treated patients withdrew consent due to treatment-related AEs [12]. The percentage of overall AEs in our study compared well to the NETTER-1 cohort (98% *vs*. 95%). However, we observed only milder symptoms, grades 1 and 2 while the NETTER-1 study reported 41% of AEs were grades 3 or 4, including severe neutropenia 1%, thrombocytopenia 2%, and lymphopenia 9% [12].

In comparison with other studies with higher administered activity, our study showed better performance with fewer AEs [12-15, 20]. Study in smaller groups of patients with pheochromocytoma/ paragangliomas [17] and rectal NET [18], though showed no significant toxicity like ours, however, we recruited a larger number and more diverse primary NETs in our study.

Indeed, studies reported up to 2% of patients developed myelodysplastic syndrome when treated with ^177^Lu-DOTATATE [20, 21]. Table 8 compares cumulative activity and AEs in our study with other trials.

The reason for no severe AEs in ours is likely related to the tailoring of administered activity to the symptoms and overall fitness of the individual.

The administered activity of ^177^Lu-DOTATATE can potentially lead to significant irradiation of the kidneys. By comparing the results of iGFR to that eGFR obtained using CKD-EPI, MDRD, and CrCl methods, the best correlation was found in the CKD-EPI and then MDRD methods. Most of our participants have a component of renal dysfunction at baseline. This added radiation exposure places the kidneys at high risk for dysfunction. Dynamic protocols with lower administered activities and an accurate assessment of GFR may create the opportunity for further management of the patient with 177Lu-PRRT therapy beyond 4 induction cycles.

Our study with a dynamic protocol of administered activity modification had better performance with less impact on bone marrow and renal function. In comparison with a study by Bodei *et al.* [15] with higher cumulative activity, our study showed a milder reduction in blood indices with 5%, 29%, and 9% reduced Hgb, WBC, and Plt, compared to 8%, 28%, and 20% respectively. Regarding CrCl, our cohort demonstrated better results with 8.8% reduced CrCl compared to 21.7% from their study after 6 months [15].

Our data showed abnormalities in eGFR and blood index more commonly occurred in the second half of the post-therapy cycle so, we would suggest focusing blood sampling toward the end of the 8-week window between treatment cycles. Multiple blood samplings are not only uncomfortable but may put additional economic hardships on patients, such as the cost of travel, and the time off work.

In our study, the first ^177^Lu-DOTATATE dose has been chosen based on eGFR and hematologic indices at baseline, the following ^177^Lu-DOTATATE administered activity was tailored to the patient's unique clinical circumstances based on the eight weeks lab data post first treatment rather than a fixed-dose. This led to a mild variation of biomarker values during the 36 weeks of induction therapy. Treatment cycles were delayed if a reduction in eGFR, biochemical abnormalities, or alarming symptoms were observed. Since the therapeutic benefit of ^177^Lu-DOTATATE takes place over months, delaying a cycle by a few days or weeks is unlikely to impact the patient's prognosis negatively.

All participants were considered as a candidate for maintenance therapy if the disease remained stable or responded to induction therapy by imaging and demonstrated no stable or late toxicity in their blood work. The blood tests at six months following completion of induction therapy (before maintenance therapy) are considered as the indicator of late or sustained toxicity. Blood tests at this time point demonstrated overall stability or recovery of Hgb, Plt, WBC, and neutrophil counts, Cr, and eGFR values. The liver enzyme demonstrated a mild increase at a 6-month time point after the fourth cycle of treatment. It may be partly explained by metastatic disease to the liver, given (62%) of our participants had evidence of liver metastases as well as undiagnosed underlying liver dysfunction secondary to drugs, obesity, or cirrhosis.

Our protocol also was considerably more efficient in various types of NET primaries with longer progression-free survival as demonstrated in the efficacy part of this trial which has recently been published [22]. The effectiveness and lack of life-threatening AEs reported in multiple clinical trials which confirmed in our study make PRRT therapy an attractive and well-tolerated option for metastatic and progressive disease [12-18, 23-25].

Even though, that available data regarding the safety of ^177^Lu-DOTATATE supports a treatment protocol with larger fixed administered activity up to 7.4 GBq (200 mCi) per cycle and including patients with lower GFR or hematologic indices in the treatment plan than our study [26], our modified dose administration protocol with fewer side effects and better outcome can be considered as a good alternative. 

## CONCLUSION

Limitations: No dosimetry was done in this study, and the administered activity was modified based on participant symptoms and blood counts, renal and liver toxicities; however, no severe toxicity in this study supported the safety of the current therapy protocol. There was no control arm in the study to compare symptoms, ECOG PS, hematologic, liver, and renal function abnormalities before, during, and at the end of treatment. Again, our data were comparable to other trials from the safety perspective without reported severe toxicity. Finally, the study sample size was average among the clinical trials in this field. The reason was potentially due to a single-center study of a relatively rare primary tumor. Notwithstanding, our data demonstrated better results of PRRT response in a variety of primary NETs with no high-grade AEs, and comparable low-grade AEs compared to others with the same or larger-scale studies.

In summary, this study reports an improvement in the severity of AEs using a dynamic administered activity protocol for ^177^Lu-DOTATATE therapy and reinforces the overall safety and generalizability of ^177^Lu-PRRT therapy to other NETs.

The number of patients with symptoms is similar to literature values, except that all AEs were mild and self-limiting, and no patient developed a severe or life-threatening AEs. Most patients tolerated this protocol well and were capable of completing induction therapy. Treatment-related changes to hematologic indices and biomarkers are predictable. Critical differences in blood work are best seen four and eight weeks after a cycle of treatment. By targeting blood work closer to the next scheduled therapy, fewer laboratory visits may be necessary. This study also shows that blood work can reliably measure renal function (eGFR) using either CKD-EPI or MDRD methods. As NETs are typically a more indolent disease, patients are at risk of over-aggressive management. Further research into the importance of patient characteristics in protocol development is necessary.

## Figures and Tables

**Fig. (1):**
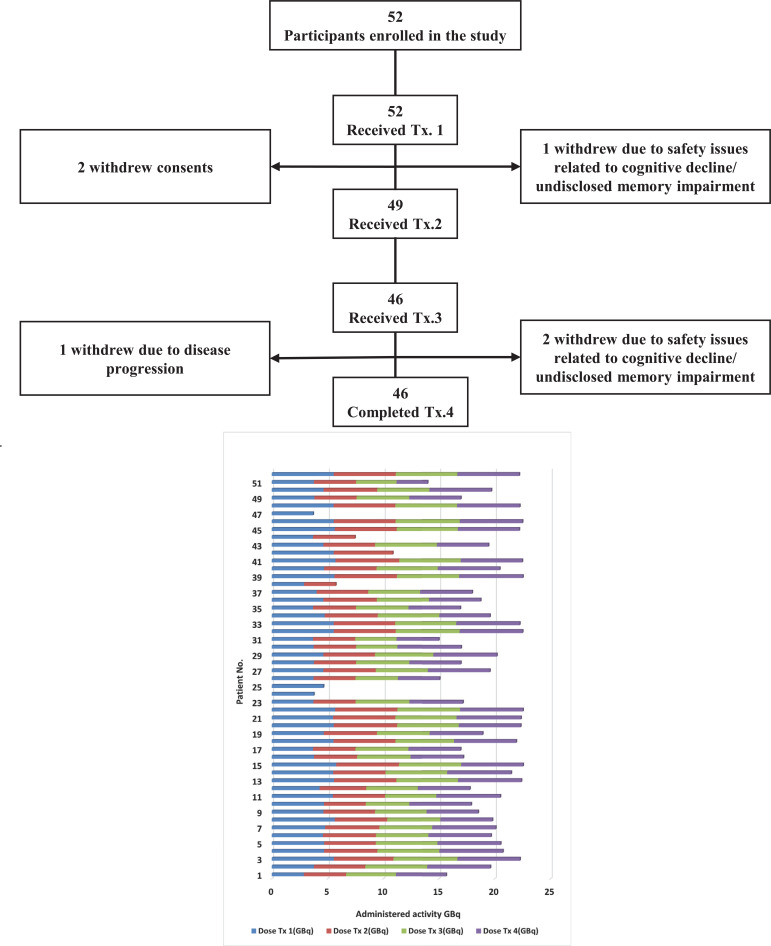
Swimmer plot representing cumulative and per cycle administered activity of the cohort.

**Fig. (2):**
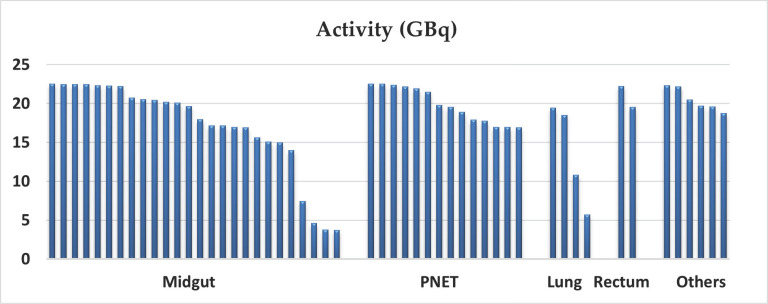
The cumulative administered activity of different primary NETs.

**Table 1:** Inclusion and exclusion criteria.

**Including Criteria**	**Excluding Criteria**
An adult patient with SSRT positive well-differentiated disease by Ki-67 < 20%	any curative surgery, radiation therapy, radioisotope therapy, change in Sandostatin LAR therapy dosage, cytotoxic chemotherapy, embolization, or other investigative therapy (interferons, mTOR inhibitors) within 12 weeks of enrollment
Positive octreotide scan (Octreoscan) with Krenning score of 2 or higher within 16 weeks of enrollment	
life expectancy greater than 26 weeks from enrollment	known brain metastases unless these metastases have been treated and/or are stable (confirmed by CT) for ≥ 24 weeks before enrollment
Cr ≤ 130 μmol/L, CrCl GFR ≥ 50mL/min/1.73m^2^ measured within 4 weeks of enrollment,	
Hgb ≥ 90 g/L; WBC ≥ 3 x 10^9^/L; absolute neutrophil count >1.5x10^9^/L; Plt ≥ 100 x 10^9^/L measured within 4 weeks of enrollment	uncontrolled diabetes mellitus, other significant medical, psychiatric, or surgical condition uncontrolled by treatment, which may interfere with completion or conduct of the study
ECOG Score ≤ 2 measured within 4 weeks of enrollment,	
written informed consent before enrollment	pregnancy and breastfeeding.

**Table 2:** Grade 3 and 4 adverse events definitions based on CTCAE 4.03.

**Biomarkers**	**Grade 3 Severe AEs**	**Grade 4 Life-threatening AEs**
**Hgb**	< 80 g/L needs transfusion	Life-threatening conditions need urgent intervention
**WBC**	< 2.0 - 1.0 x 10^9^ /L	< 1.0 x 10^9^ /L
**Neutrophil**	< 1.0 - 0.5 x10^9^ /L	< 0.5 x 10^9^ /L
**Plt**	< 50.0- 25.0 x 10^9^ /L	< 25.0 x 10^9^ /L
**Cr**	>3.0 baseline; >3.0 - 6.0 x ULN	>6.0 x ULN
**eGFR**	29 - 15ml/min/1.73 m^2^	<15 ml/min/1.73m^2^; dialysis or renal transplant needed
**ALT**	>5.0 - 20.0 x ULN	>20.0 x ULN
**AST**	>5.0 - 20.0 x ULN	>20.0 x ULN
**ALP**	>5.0 - 20.0 x ULN	>20.0 x ULN

AEs: adverse events, Hgb: hemoglobin, WBC: white blood cells, Plt: platelets, Cr: creatinine, eGFR: estimated glomerular filtration rate, ALT: alanine aminotransferase, AST: aspartate aminotransferase,ALP: alkaline phosphatase.

**Table 3:** 177-Lu- DOTA-TATE administered activity modifications

**Risk Factor**	**Dose**				**Notes**
	**150 mCi** **(5.55 GBq)**	**125 mCi** **(4.62 GBq)**	**100 mCi** **(3.7 GBq)**	**75 mCi** **(2.78 GBq)**	
Age (years)	< 65	65-75	> 75	If more than 2 risk factors identified.	-
Glomerular filtration rate	≥ 65 corrected	56-65 corrected	50-55 corrected		< 50 corrected: Tx not offered.
Liver disease involvement (CT/MRI/^177^Lu scan) and increased liver enzymes	≤ 70% of liver involved approximately		> 70% of liver involved		Enzymes > 3 × normal limits: Tx not offered.
Platelets	≥ 100×10^9^/L	≥ 100×10^9^/L	≥ 100×10^9^/L		< 100×10^9^/L (< 95×10^9^/L for subsequent Tx).
White blood cells	≥ 3×10^9^/L	≥ 3×10^9^/L	≥ 3×10^9^/L		ANC< 1.5 ×10^9^/L: Tx not offered.
Previous nephron- and/or marrow- toxic therapies	None	1 course of chemotherapy or ≤ 4 RIT with dose < 800 mCi.	>1 course of chemotherapy or > 4 RIT with dose > 800 mCi.		-
Weight(kg)	≥ 60	50-59	<50		Dose to be not > 2.5 mCi/kg (0.07 GBq/kg)
Bone and marrow disease involvement of axial skeleton	≤ 5 bone metastases	6-10 bone metastases	>10 bone metastases	Diffuse bone marrow involvement	-

**Table 4:** Demographic and baseline characteristics.

**Sex (M, F)**	**32 (62%), 20 (38%)**
Mean Age at Diagnosis (years)	57
Mean Age at Enrollment (years)	62.5
Primary Tumor	-
GNET (Small Bowel /distal ileum)	26 (50%)
PNETs	14 (27%)
Bronchial NET	4 (8%)
Rectal/ Rectosigmoid NET	2 (4%)
Pheochromocytoma	1 (2%)
Eustachian tube	1 (2%)
Renal	1 (2%)
Ovarian	1 (2%)
Thymic NET	1 (2%)
Unknown	1 (2%)
Mean Ki-67Tumour Grade	-
Grade 1	24 (46%)
Grade 2	28 (54%)
Mean Baseline Chromogranin A	-
≤110	16 (31%)
>110	36 (69%)
ECOG Performance Status	-
0	20 (38%)
1	29 (56%)
2	3 (6%)

GNET: Gastrointestinal Neuroendocrine Tumors, PNETs: Pancreatic Neuroendocrine Tumors, ECOG: Eastern Cooperative Oncology Group. Chromogranin A measurement: CIS BIO Chromogranin A ELISA with reference range <= 110 ng/mL.

**Table 5:** Reported symptoms and ECOG score before and following PRRT.

**Symptoms**	**Baseline before the Study (%)**	**Symptom Relieved**	*New **from Baseline**	**EOT Symptoms Including Baseline (%)**
**ECOG** ≥ **1**	32 (62)	8	10	35(67)
**Fatigue**	31 (60)	7	16	32 (62)
**Shortness of breath**	18 (35)	2	12	26 (50)
**Nausea**	14 (27)	3	10	23 (44)
**Abdominal pain**	17 (33)	4	7	20 (38)
**MSK pain**	15 (29)	2	8	19 (37)
**Loss of appetite**	17 (33)	1	4	18 (35)
**Diarrhea**	13 (25)	3	6	16 (31)
**Flushing**	10 (19)	2	7	15 (29)
**Headache**	4 (8)	0	9	13 (25)
**Edema**	3 (6)	1	4	6 (12)
**Hair loss**	3 (6)	0	5	6 (12)
**Rashes**	1 (2)	1	5	6 (12)
**Cough**	2 (4)	1	5	6 (12)
**Vomiting**	1 (2)	1	4	4 (8)
**Distention**	2 (4)	1	1	2 (4)
**Dizziness**	0 (0)	51	2	2 (4)
**All clinical adverse events AE**	42 (81)	1	9	50 (96)

**Notes:** Patients were considered as their control. This Data set was collected from clinic notes and the Edmonton Scale Assessment System (self-reported by patients). * Symptoms seen during the trial and at the end of the study. EOT: End of Treatment.

**Table 6:** Hematologic, hepatic, and renal indices before administration of and following PRRT.

**Biomarker**	**Before Trial (Baseline)**		**Treatment 4 (EOT)**	
	**No AE %**	**Any Grade AE %**	**No AE %**	**Any Grade AE %**
**GFR**	54	46	42	58
**Cr**	92	8	85	15
**Hbg**	46	54	19	81
**WBC**	94	6	63	37
**Neutrophil**	96	4	88	12
**PLT**	88	12	75	25
**ALT**	96	4	77	23
**ALP**	75	25	50	50
**AST**	94	6	76	19
**Any above AE**	17	83	2	98

**Abbreviations:** GFR: Glomerular filtration rate, Cr: creatinine, Hgb: hemoglobin, PLT: platelets, WBC: white blood cells, ALT: alanine aminotransferase, AST: aspartate aminotransferase, ALP: alkaline phosphatase, EOT: End of treatment.

**Table 7:** Adverse events reported in the current study and the NETTER-1 trial

**Symptoms**	**Current Study %**	**NETTER-1%**
**Fatigue**	62	40
**Shortness of breath**	50	N/A
**Nausea**	44	59
**Abdominal pain**	38	26
**MSK pain**	37	32
**Loss of appetite**	35	18
**Diarrhea**	31	29
**Flushing**	29	13
**Headache**	25	16
**Edema**	12	14
**Alopecia**	12	11
**Rashes**	12	N/A
**Cough**	12	11
**Vomiting**	8	47
**Dizziness**	4	11
**Distention**	4	13

**Table 8:** comparison of cumulative activity and AEs between studies.

**Study/Refs.**	**No. of Cases** **(Type of NETs)**	**Cumulative** **(Cycle) Activity GBq**	**% of Grade 3-4 Biomarkers and other AEs**
**NETTER’s **[12]	111 (midgut)	29.6 (7.4)	41%; majority GI, MSK and general symptoms and includes 2% thrombocytopenia, 1% neutropenia, 1% leukopenia and 9% lymphopenia
**Garske-Román** [13]	200 (PNET, rectal, lung, unknown)	29.6 (7.4)	15% BMT
**Kwekkeboom** [14]	504 (carcinoid, PNET and unknown primary NETs)	27.8-29.6 (3.7-7.4)	0.8% MDS, 9.5% hematological, 1.2% hospitalized due to hormone crisis
**Bodei** [15]	51 (variety)	Two group with median cumulative activity 25.2-26.4 (escalating activity from 3.7-5.18 in group 1 and 5.18-7.4 in group 2)	2% leukopenia with thrombocytopenia
**Vyakaranam ** [17]	22 (paragangliomas)	29.6 (7.4)	0%
**Kong** [18]	27 (rectal)	30 (7.4)	Late grade 3 lymphopenia in 11% at 3 months Post PRRT
**Current study**	52 (variety)	19.59 (2.78-5.5)	0%
**Bodei** [20]	290 (Gastroenteropancreatic, bronchial and unknown)	23.3	0% renal, 3.1% hematological toxicity

**Abbreviations:** NETs: neuroendocrine tumors, GI: gastrointestinal, MSK: musculoskeletal, PNET: pancreatic neuroendocrine tumor, MDS: myelodysplastic syndrome, BMT: bone marrow toxicity, PRRT: peptide receptor radionuclide therapy.

## LIST OF ABBREVIATIONS

Lu: Lutetium
PRRT: Peptide Receptor Radionuclide Therapy
NETs: Neuroendocrine Tumors
AEs: Adverse Events
GBq: Gigabequerel
mCi: Millicurie
eGFR: Estimated Glomerular Filtration Rate
ALP: Alkaline Phosphatase
SSTR: Somatostatin Receptor
SSA: Somatostatin Analogue
SSRT: Somatostatin Receptor
Cr: Creatinine
CrCl GFR: Creatinine Clearance Glomerular Filtration Rate
Hgb: Hemoglobin
WBC: White Blood Cells
Plt: Platelets
ALT: Alanine Aminotransferase
AST: Aspartate Aminotransferase
ECOG PS: Eastern Cooperative Oncology Group Scale Performance Status
RFA: Radiofrequency Ablation
mCi: Millicurie
ESAS: Edmonton Symptom Assessment System
CTCAE: Common Terminology Criteria for Adverse Events
MDRD: Modification of Diet in Renal Disease
CKD-EPI: Chronic Kidney Disease Epidemiology
iGFR: Isotope GFR
DTPA: Diethylenetriamine Penta acetic Acid
PNET: Pancreatic Neuroendocrine Tumor
SPECT/CT: Single-Photon Emission Computed Tomography/ Computed Tomography
EOT: End of Treatment
MSK: Musculoskeletal
MDS: Myelodysplastic Syndrome
BMT: Bone Marrow Toxicity

## AVAILABILITYOFDATAANDMATERIALS

Thedatasets usedand/or analysed duringthecurrent study are available fromthecorresponding author [A.K.] on reasonable request.
